# Silver(I)-Tazobactam Frameworks with Improved Antimicrobial Activity

**DOI:** 10.3389/fchem.2021.815827

**Published:** 2022-01-25

**Authors:** Daniela R. Ferreira, Paula C. Alves, Alexander M. Kirillov, Patrícia Rijo, Vânia André

**Affiliations:** ^1^ Centro de Química Estrutural, Instituto Superior Técnico, Universidade de Lisboa, Lisboa, Portugal; ^2^ Associação do Instituto Superior Técnico para a Investigação e Desenvolvimento (IST-ID), Lisboa, Portugal; ^3^ Universidade Lusófona’s Research Center for Biosciences and Health Technologies (CBIOS), Lisboa, Portugal; ^4^ Faculty of Pharmacy, Research Institute for Medicines (iMed. ULisboa), Universidade de Lisboa, Lisboa, Portugal

**Keywords:** mechanochemistry, supramolecular chemistry, tazobactam, silver, antibiotic coordination frameworks

## Abstract

Tazobactam (TazoH) is a penicillinate sulfone β-lactamase inhibitor with negligible antimicrobial activity, commonly used with other antibiotics to provide an effective combination against many susceptible organisms expressing β-lactamases. Two novel Ag(I)-tazobactam frameworks ([Ag(I)-Tazo] and [Ag(I)-Tazo_2_]) prepared by mechanochemistry are presented herein as alternative forms to improve the antimicrobial activity of tazobactam by exploring synergistic effects with silver, being the first crystal structures reported of tazobactam coordinating to a metal site. The topological analysis of the 3D ([Ag(I)-Tazo]) and 2D+1D ([Ag(I)-Tazo_2_]) frameworks revealed underlying nets with the **cbs** (CrB self-dual) and decorated **sql** topologies, respectively. These novel frameworks are stable and show an enhanced antimicrobial activity when compared to tazobactam alone. Amongst the tested microorganisms, *Pseudomonas aeruginosa* is the most sensitive to tazobactam and the new compounds. This study thus unveils novel facets of tazobactam chemistry and opens up its application as a multifunctional linker for the design of antibiotic coordination frameworks and related materials.

## Introduction

The development of antibiotics was one of the most significant medical achievements in the treatment of microbial infections, and therefore has saved numerous lives. Nevertheless, the inappropriate use of antibiotics is one of the causes of the emerging antimicrobial resistance (AMR), which represents a major threat to modern society and makes most of the commercially available antimicrobial drugs ineffective (Tooke et al., 2019).

Amongst the different classes of antibiotics, β-lactams encompass several of the most used antimicrobial agents, such as penicillins, cephalosporins, carbapenems and monobactams. The first β-lactam antibiotic, penicillin G, was developed to treat bacterial infections. As these compounds affect the biosynthesis of the bacterial cell wall and demonstrated high efficiency, there has been an increase in the search, development and production of similar penicillin derivatives (Drawz and Bonomo 2010; Bozcal and Dagdeviren 2017; Tooke et al., 2019). In particular, the emergence of β-lactamase-mediated resistance can compromise β-lactam antibiotics efficiency. One of the strategies explored to circumvent this problem concerns the development of selective β-lactamase inhibitors for co-administration with β-lactam antibiotics (Drawz and Bonomo 2010; Docquier and Mangani 2018).

**FIGURE 1:**
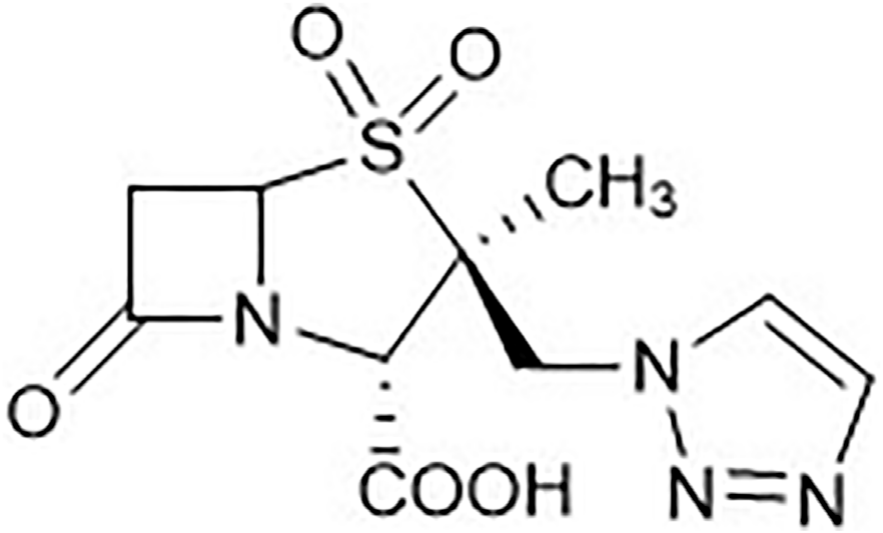
Tazobactam (TazoH) representation.

Tazobactam (Figure 1) is an effective inhibitor of β-lactamase against several susceptible organisms expressing various classes of β-lactamases (Fisher et al., 1980; Drawz and Bonomo 2010). It is a penicillinate sulfone, with similarities to the structure of penicillin, however when used as a drug alone it has low antibacterial activity. This compound has been widely used in combination with β-lactam antibiotics in clinical use (Drawz and Bonomo 2010). One of the latest combination therapies with tazobactam relies on its combination with ceftolozane, an antipseudomonal cephalosporin.

Frequently, patients susceptible to Gram-negative bacilli, such as *Pseudomonas aeruginosa* and *Enterobacterales*, are diagnosed with bacterial pneumonia. The therapeutic combination ceftolozane/tazobactam is widely used in the treatment of multidrug-resistant (MDR) *P. aeruginosa* infections. This Gram-negative bacterium is an especially important nosocomial pathogen with intrinsic resistance to several classes of antimicrobial agents (Jacqueline et al., 2017; Sheffield et al., 2020; Karlowsky et al., 2021). Another possible therapy combines piperacillin and tazobactam (P/T) which is used in the treatment of *P. aeruginosa* and methicillin-resistant *Staphylococcus aureus* (MRSA) (Asai et al., 2021). This P/T combination is recommended for clinical use by the World Health Organization Model List of Essential Medicines [22^nd^ List (2021)] as the first choice for severe intra-abdominal infections, high-risk febrile neutropenia, acquired pneumonia at the hospital and necrotizing fasciitis (World Health Organization 2021).

In order to face the microbial (multi) resistance to drugs/antibiotics, several approaches have been developed and tested to augment or improve the arsenal of tools to battle this health growing problem. One of the strategies that could lead to considerable results is the application of nanoporous materials due to their high specific surface area and correlations between the structure and surface characteristics. Metal-organic frameworks (MOFs) belong to this family of materials and represent one of the most current advances in coordination chemistry and supramolecular engineering (Tanaka 2020). MOFs are hybrid extended networks derived from inorganic units (as isolated cations, chains, clusters) and organic polydentate ligands, building a flexible family of crystalline compounds with an extraordinary variety of applications in chemical and material sciences (Rojas et al., 2019), including gas storage (Qasem et al., 2018), catalysis (Hong et al., 2021), luminescence (Huang et al., 2019; Liu et al., 2021a), electrochemistry (Wen et al., 2012; Wang et al., 2020), and separation (Kadioglu and Keskin 2018). MOFs with biomedical and pharmaceutical properties have been reported as carriers for the controlled delivery of different active species (Mileo et al., 2021), for cutaneous and cosmetic treatments (Duan et al., 2021), and as contrast agents for magnetic resonance imaging (Zhang et al., 2018; Hu et al., 2021), not disregarding the potential biomedical/pharmaceutical applications in cancer (Zhang et al., 2018; Li et al., 2020; Liu et al., 2021b; Liu et al., 2021c) and antimicrobial (André et al., 2021; Liu et al., 2021d) therapies.

MOFs based on bioactive components (BioMOFs) are an emerging subclass of MOFs, constructed from a variety of metals and ligands such as amino acids, peptides, nucleobases, saccharides, active pharmaceutical ingredients, and enzyme inhibitors (Rojas et al., 2017; An et al., 2019; Cai et al., 2019; Quaresma et al., 2020). Due to the low steric hindrance, low toxicity, and good biocompatibility of bioligands, BioMOFs are currently widely studied for possible applications in several areas including biomedicine (Sun et al., 2020) and biocatalysis (Celik et al., 2018). Within BioMOFs, the approach to use antibiotics for the direct coordination to biocompatible metal centers, giving rise to antibiotic coordination frameworks (ACFs), has been demonstrated to be a valid strategy to increase the efficiency of already commercially available antibiotics against some microorganisms (André et al., 2019; Quaresma et al., 2021).

Given a virtually unexplored coordination chemistry of tazobactam, the main goal of the present study consisted in probing its potential for the generation of new ACFs. Hence, two novel Ag(I)-tazobactam frameworks, [Ag(I)-Tazo] and [Ag(I)-Tazo_2_], were synthesized by mechanochemistry, a “green” synthetic route that has already shown its potential for such type of synthesis (Friščic et al., 2020; Zábranský et al., 2021). To the best of our knowledge, the obtained compounds are the first crystal structures reporting the coordination of tazobactam to metal sites. The choice of silver as a cation in these studies lies in its well-recognized antibacterial action (Alexander 2009), allowing use to be made of potential synergistic effects between both ACFs components. The ultimate goal of this study was to improve the antimicrobial activity of tazobactam from the synergy with silver, since this drug is frequently used in the treatment of bacterial infections. Hence, the synthesis, full characterization, structural features, and antimicrobial activity of the obtained ACFs, [Ag(I)-Tazo] and [Ag(I)-Tazo_2_], are described in the present work.

## Results and Discussion

Two new Ag(I)-tazobactam coordination frameworks [Ag_2_(µ_3_-Tazo)(µ_4_-Tazo)]_n_ (abbreviated as [Ag(I)-Tazo]) and {[Ag(µ-Tazo)(µ-TazoH)][Ag(µ-Tazo)(TazoH)]}_n_ (abbreviated as [Ag(I)-Tazo_2_]) were synthesized from tazobactam (TazoH) and silver(I) oxide, using 1:1 and 1:2 Ag:tazobactam ratios.

Despite the successful synthesis of both compounds by different methods, liquid-assisted grinding (LAG) performed in a vibrational ball mill was the preferred procedure, due to the low amount of solvent required (*η* = 0.5 μL/mg) (Do and Friščić 2017), a reduced reaction time, and an ease of the process without compromising high purity and yield.

Powder X-ray Diffraction (PXRD) analysis confirmed the purity of product samples obtained via the slurry reaction, manual grinding, and ball milling methods by comparing the diffractograms with those simulated from the single crystal data of [Ag(I)-Tazo] and [Ag(I)-Tazo_2_] (Supplementary Figures S1, S2). The purity of both compounds was also confirmed by elemental analysis: [Ag(I)-Tazo]: C, 29.5% calc/29.73% found; H, 2.72% calc/found 2.35%; N, 13.76% calc/13.69% found; S, 7.87% calc/8.08% found; [Ag(I)-Tazo_2_]: C, 34% calc/34.39% found; H, 3.14% calc/2.98% found; N, 15.86% calc/15.82% found; S, 9.08% calc/9.16% found.

The structural elucidation, and the physicochemical characterization are presented and discussed for both compounds, [Ag(I)-Tazo] and [Ag(I)-Tazo_2_]. Antimicrobial assays were also carried out to prove the enhanced antimicrobial activity of both compounds unveiled herein.

### Structural Characterization

**FIGURE 2:**
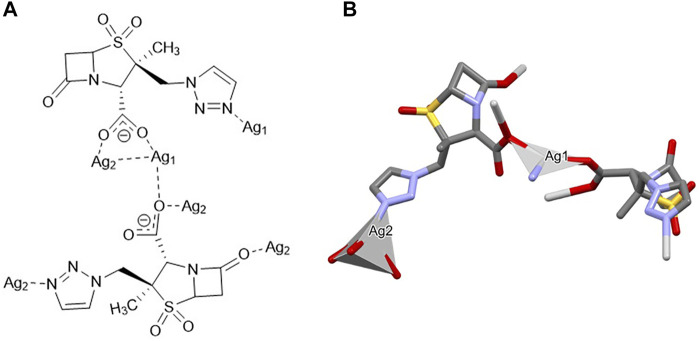
Schematic representation of **(A)** the coordination sites and **(B)** the coordination geometry around the Ag(I) sites (hydrogen atoms have been omitted for clarity reasons) for [Ag(I)-Tazo].

[*Ag*_*2*_(*µ*_*3*_ *-Tazo*)(*µ*_*4*_ *-Tazo*)]_*n*_ {[Ag(I)-Tazo]}. Despite the 1:1 ratio, the asymmetric unit of [Ag(I)-Tazo] is formed by two crystallographically independent deprotonated tazobactam moieties (µ_3_-and µ_4_-Tazo⁻) and two Ag(I) sites, Ag1 and Ag2. The µ_3_-Tazo⁻ ligand coordinates to two Ag1 sites via the carboxylate [2.229 (5) Å] and the triazole [2.281 (5) Å] functionalities, and to one Ag2 via the other O of the carboxylate group [2.297 (5) Å]. The µ_4_-Tazo⁻ coordinates to Ag1 via the carboxylate group [2.263 (5) Å] and to three different Ag2 via the other O of the carboxylate [2.607 (5) Å], the carbonyl [2.627 (5) Å] and the triazole [2.276 (5) Å] moieties. The Ag sites assume distorted trigonal planar [Ag1, NC = 3, Ag1 deviation from the plane of 0.196 (4) Å] and tetrahedral (Ag2, NC = 4) coordination geometries (Figure 2).

**FIGURE 3:**
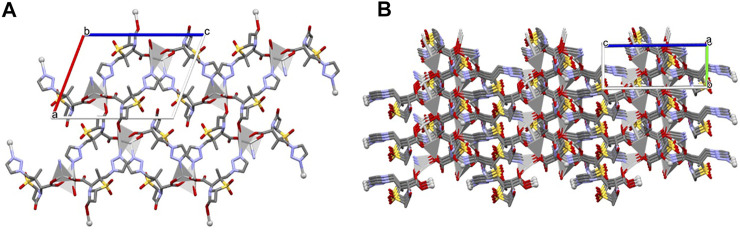
Crystal packing of [Ag(I)-Tazo], depicting **(A)** the view along the *b* axis, and **(B)** the formation of the 3D metal-organic framework.

The presence of different coordination sites gives rise to an extended 3D non-porous metal-organic framework (Figure 3), with the consecutive alternation of Ag1 and Ag2 metal sites via the µ_3_-and µ_4_-Tazo⁻ linkers. Even though no classical hydrogen bonds are present in the supramolecular arrangement of [Ag(I)-Tazo], two S-O⋅⋅⋅π [2.780 (6) Å, 60.2 (2) ⁰ and 3.545 (6) Å, 83.9 (2) ⁰] and five π⋅⋅⋅π interactions [3.681(4)—4.813(5) Å] are responsible for the interactions within the framework (Supplementary Table S2), resulting in a high packing efficiency (74.2%).

**FIGURE 4:**
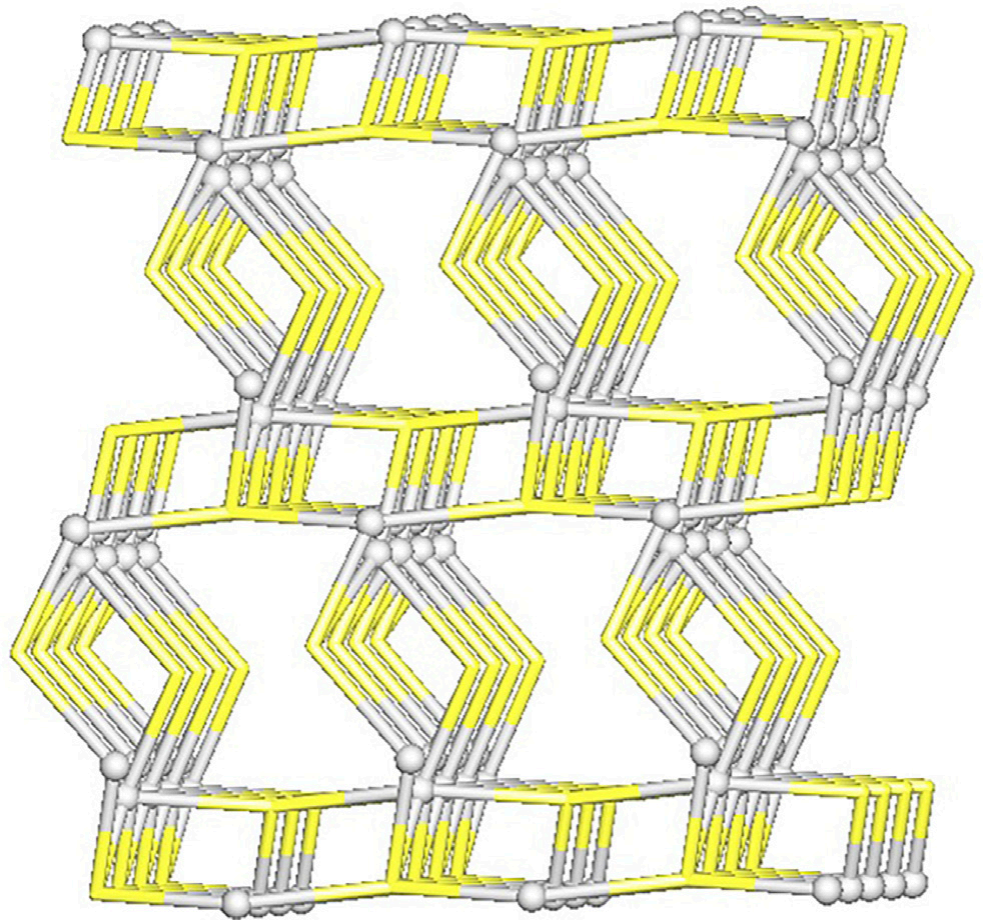
Topological representation of a dinodal 3,4-linked net in [Ag(I)-Tazo] with a **cbs** (CrB self-dual) topology; rotated view along the *b* axis; Ag1/Ag2 centers (gray balls), centroids of µ_3_-and µ_4_-Tazo^−^ moieties (yellow).

From the topological analysis, it is possible to conclude that the 3D metal-organic net is built from the 3 and 4-connected Ag1/Ag2 nodes, and the 3 and 4-connected µ_3_-and µ_4_-Tazo^−^ nodes (Figure 4). The resulting underlying network can be classified as a dinodal 3,4-linked net with a **cbs** (CrB self-dual) topology and a point symbol of (6^3^) (6^6^).

**FIGURE 5:**
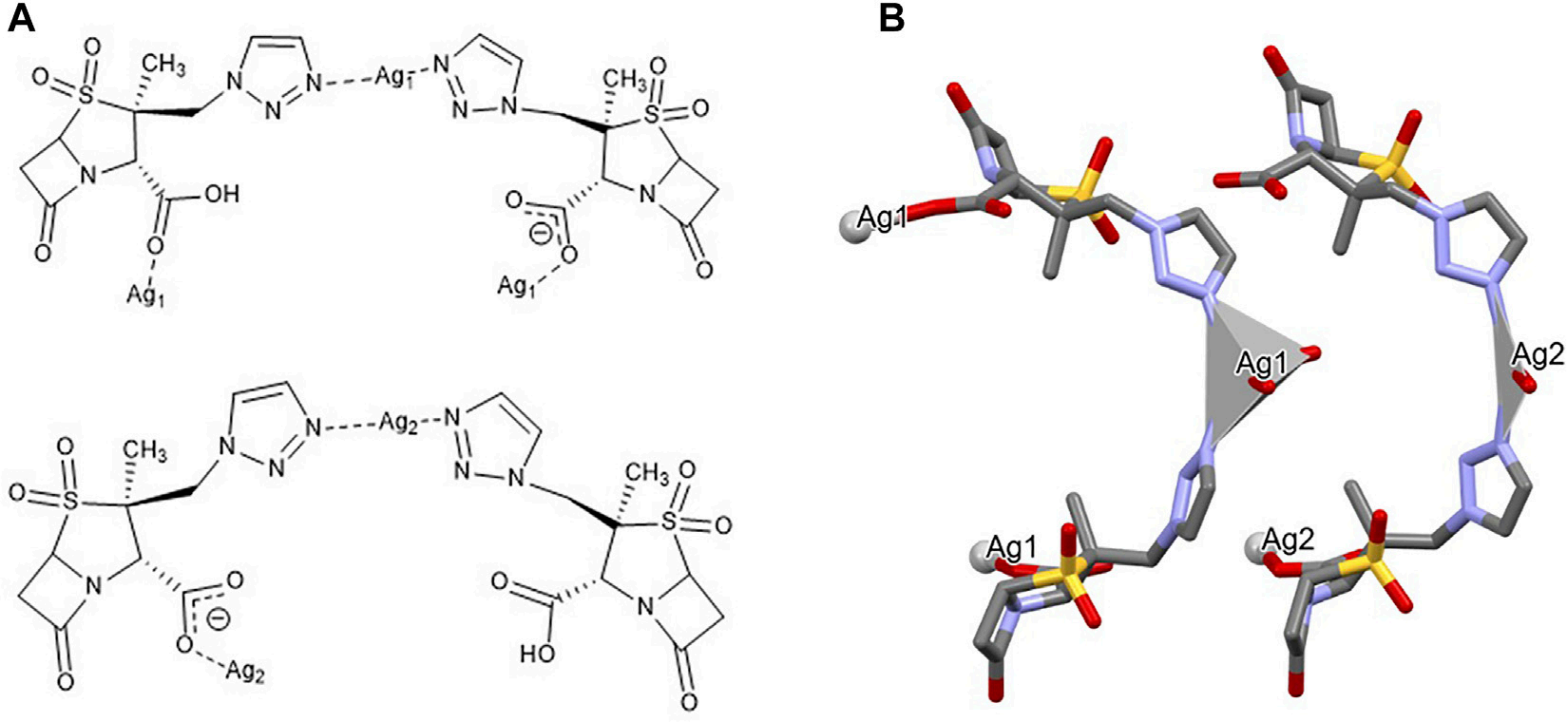
Schematic representation of **(A)** the coordination sites and **(B)** the coordination geometry around the Ag(I) sites (hydrogen atoms have been omitted for clarity reasons) for [Ag(I)-Tazo_2_].

{[Ag(µ-Tazo)(µ-TazoH)][Ag(µ-Tazo)(TazoH)]}_n_ {[Ag(I)-Tazo_2_]}. The asymmetric unit of [Ag(I)-Tazo_2_] is formed by two crystallographically independent tazobactam molecules (TazoH), two deprotonated tazobactam anions (µ-Tazo⁻) and two Ag(I) centers, fulfilling a 1:2 stoichiometry. Both Ag1 and Ag2 centers are bound by two triazole moieties [2.208 (8) and 2.209 (8) Å for Ag1, 2.168 (9) and 2.176 (9) Å for Ag2]. Ag1 is further coordinated by two carboxylate groups from µ-Tazo⁻/µ-TazoH [2.435 (7) and 2.441 (7) Å]. The Ag2 is only coordinated by the carboxylate group one µ-Tazo⁻ linker (2.504 (8) Å). Despite the different coordination patterns in [Ag(I)-Tazo_2_], the Ag sites also adopt distorted trigonal planar (Ag1, NC = 3) and tetrahedral (Ag2, NC = 4) coordination geometries (Figure 5).

**FIGURE 6:**
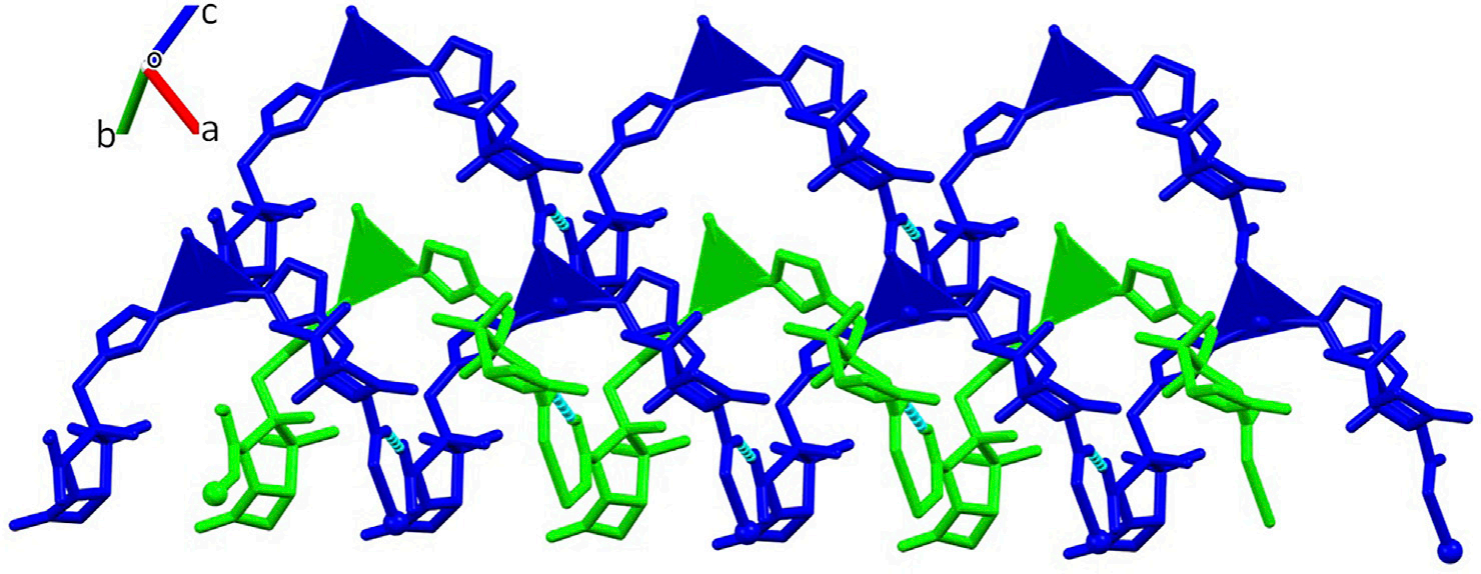
Crystal packing of [Ag(I)-Tazo_2_] depicting the 2D (blue) and 1D (green) coordination networks.

**FIGURE 7:**
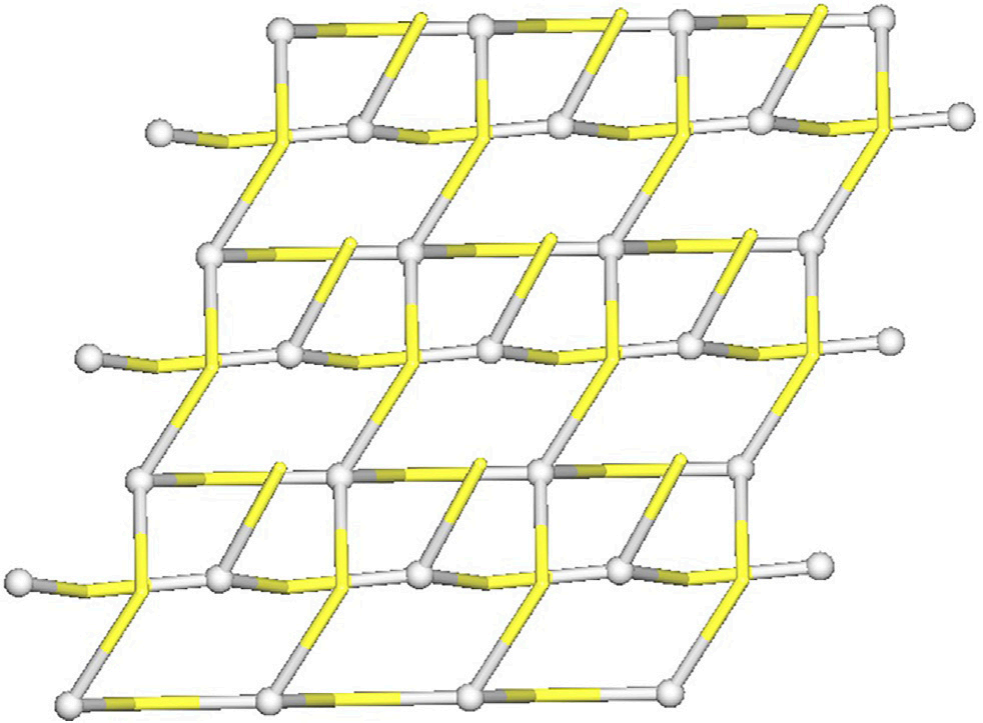
Topological representation of a mononodal 4-linked metal-organic layer in [Ag(I)-Tazo_2_] with a **sql** topology, decorated with 2C1 chains; rotated view along the *b* axis; Ag1/Ag2 centers (gray balls), centroids of TazoH/Tazo^−^ moieties (yellow).

The structure of [Ag(I)-Tazo_2_] reveals two independent 2D and 1D coordination networks (Figure 6). The 2D net is formed by the crystallographically independent µ-Tazo⁻ and µ-TazoH moieties that act as linkers for Ag1 centers. The 1D chain is based on Ag2 centers and comprises the terminal TazoH ligands and µ-Tazo⁻ linkers. An overall structure of [Ag(I)-Tazo_2_] can be defined as a 2D+1D coordination polymer wherein 1D chains (based on Ag2 atoms) are interdigitated into 2D layers (based on Ag1 atoms) (Figure 6). Each of these motifs is reinforced by hydrogen bonds between COOH/COO^−^ groups of tazobactam ligands [O–H···O 2.421 (11) and 2.469 (12) Å] (Table 1); however, there are no classical hydrogen bonds between the 1D and 2D networks. Several weaker interactions are also established (Supplementary Table S2). This structure has a packing efficiency of 69.6%. From a topological perspective, the resulting underlying network can be classified as a 4-linked **sql** [Shubnikov tetragonal plane net] layer decorated with the chains of 2C1 topology (Figure 7 and Supplementary Figure S3). The **sql** layer is composed of the 4-connected Ag1 centers and µ-Tazo^−^ linkers, leading to a (4^4^.6^2^) point symbol.

**TABLE 1:** List of the main hydrogen bonds found for compound [Ag(I)-Tazo_2_].

Sym. Op	D–H···A	*d* (D–H) (Å)	*d* (H···A) (Å)	*d* (D···A) (Å)	(DHA) (deg)
*-1+x, y, -1+z*	O_7_–H_70_···O_1_	0.82	1.71	2.421 (11)	143
*-1+x, y, -1+z*	O_17_–H_170_···O_11_	0.82	1.73	2.469 (12)	148

**FIGURE 8:**
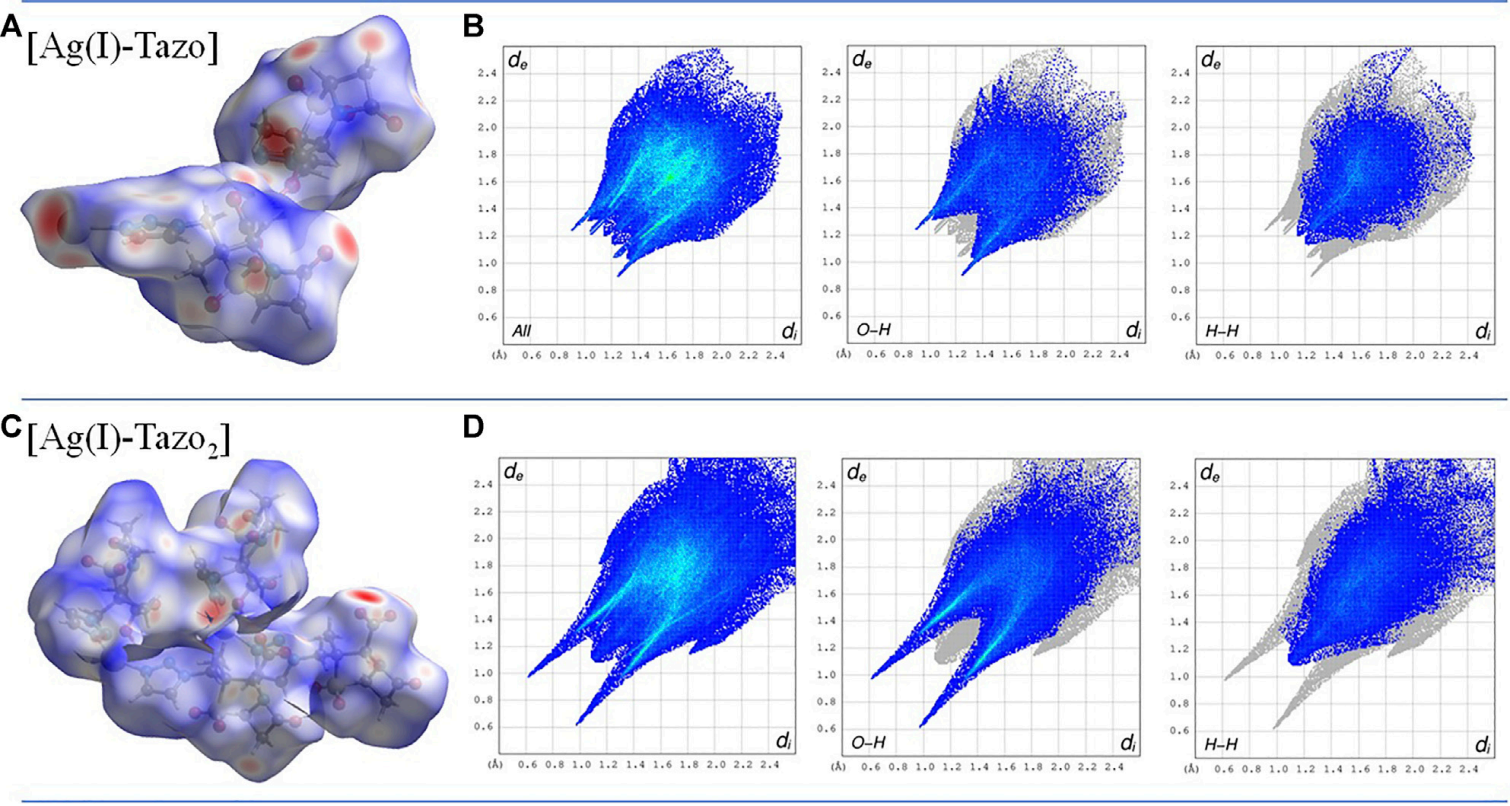
Hirshfeld surface mapped with d_norm_, with surfaces shown as transparent to allow the visualization of the compound **(A)** and two-dimensional fingerprint plots **(B)** for the most relevant interactions in [Ag(I)-Tazo] **(C)** and [Ag(I)-Tazo_2_] **(D)**.

The study of the packing interactions is complemented by the analysis of the Hirshfeld surfaces and the corresponding two-dimensional fingerprint plots (Figure 8). The O-H interactions are the most abundant in both compounds, but the presence of hydrogen bonds in [Ag(I)-Tazo_2_] is responsible for a higher percentage of this type of interactions in [Ag(I)-Tazo_2_] than in [Ag(I)-Tazo] (44.3 *vs* 36.5%, respectively). The two spikes in the 2D fingerprint plots corresponding to the O-H interactions are sharper in [Ag(I)-Tazo_2_], due to the presence of hydrogen bonds and shorter distances. The O-H interactions can be identified as red regions in the d_norm_ representation, supporting the interactions previously described. The H-H interactions appear in the middle-scattered points in the 2D fingerprint map and are the second most relevant type of interactions {23 and 28.1% for [Ag(I)-Tazo] and [Ag(I)-Tazo_2_], respectively}. The N-H interactions are also significant, representing 10.8 and 5.5% of the total Hirshfeld surface for [Ag(I)-Tazo] and [Ag(I)-Tazo_2_], respectively, as well as Ag-O {8.6 and 5.2% for [Ag(I)-Tazo] and [Ag(I)-Tazo_2_], respectively}. Apart from these, π⋅⋅⋅π (C–C) contacts are observed in [Ag(I)-Tazo] corresponding to 1.7%, but they are not present in [Ag(I)-Tazo_2_], with lone-pair⋯π (O–C) interactions being also higher in [Ag(I)-Tazo] than in [Ag(I)-Tazo_2_] (1.8 *vs* 1%). On the other hand, the lone-pair⋯lone-pair (O–O) interactions are higher in [Ag(I)-Tazo_2_], corresponding respectively to 5.6% *vs* 3% in [Ag(I)-Tazo]. All the other interactions are present in minor percentages (see supplementary material for full details, Supplementary Figures S4–S6).

FTIR-ATR analysis of tazobactam, [Ag(I)-Tazo] and [Ag(I)-Tazo_2_] (Supplementary Figure S7 and Supplementary Table S3 **)** makes evident that the stretching vibrations of the C-SO_2_-C and N=N groups are visible in TazoH (C-SO_2_-C:1140, 1190, 1313 cm^−1^; N=N: 1238, 1455 cm^−1^) as well as in both compounds [Ag(I)-Tazo] (C-SO_2_-C: 1139, 1206, 1311 cm^−1^; N=N: 1233, 1446 cm^−1^) and [Ag(I)-Tazo_2_] (C-SO_2_-C: 1142, 1188, 1321 cm^−1^; N=N: 1241, 1455 cm^−1^) without significant changes. Differences in the bands associated with the carboxylic moiety of tazobactam can be detected. The peaks concerning the carbonyl group from the amide are found at 1795, 1791 and 1773 cm^−1^ for TazoH, [Ag(I)-Tazo] and [Ag(I)-Tazo_2_], respectively. The main differences in the three spectra correspond to the peaks at around 1610 and 1380 cm^−1^, characteristic of carboxylate moieties present in both structures. The carbonyl group stretching from the carboxylic acid moiety detected at 1702 cm^−1^ in TazoH, is not seen in [Ag(I)-Tazo] due to the fully deprotonated Tazo^−^ linkers, with the characteristic peaks of the carboxylate being detected at 1373 and 1599 cm^−1^. In [Ag(I)-Tazo_2_], the protonated tazobactam gives rise to the peak at 1702 cm^−1^, while the deprotonated Tazo^−^ moiety is identified by the peaks at 1384 and 1610 cm^−1^.

### Stability of the Compounds

The stability of the compounds is a very important factor to be controlled, especially for medical/pharmaceutical applications. Considering the PXRD diffractograms of the obtained compounds monitored along time, it can be concluded that no degradation of these silver-organic frameworks under shelf storage conditions is detected, as the patterns remain unchanged for at least 5 months (Supplementary Figures S8–S13).

**FIGURE 9:**
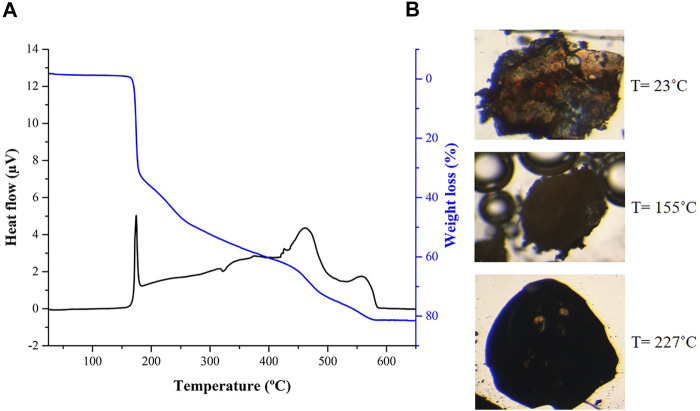
**(A)** Differential scanning calorimetry (DSC) and thermogravimetry (TGA) and **(B)** hot-stage microscopy (HSM) images at 23, 155 and 227°C for compound [Ag(I)-Tazo_2_].

Regarding thermal stability, DSC/TGA data are very similar for both [Ag(I)-Tazo] and [Ag(I)-Tazo_2_] (Figure 9 and Supplementary Figure S14), showing their stability until 155°C, temperature at which melting, and decomposition begin. These observations are confirmed by hot-stage microscopy (HSM) data.

### Antimicrobial Activity Assays

A microbial screening for the assessment of the antimicrobial activity of the new Ag(I)-tazobactam frameworks was carried out by the determination of their minimum inhibitory concentration (MIC) median values via the microdilution method (Clinical and Laboratory Standards Institute 2018; Alves et al., 2020).

**FIGURE 10:**
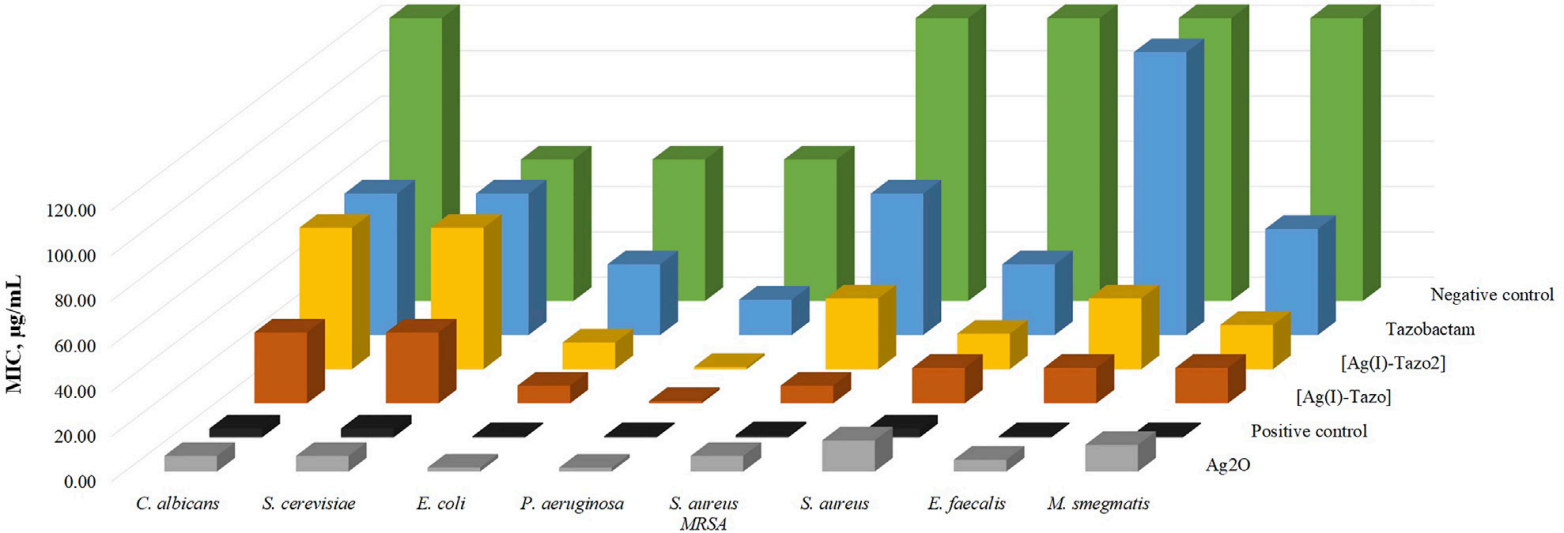
Minimum inhibitory concentration values (MIC, µg/ml) of the synthesized compounds {[Ag(I)-Tazo] and [Ag(I)-Tazo_2_]} and the starting materials for *Candida albicans* and *Saccharomyces cerevisiae* (yeasts), *Escherichia coli* and *Pseudomonas aeruginosa* (Gram-negative bacteria) and *Staphylococcus aureus*, *Enterococcus faecalis* and *Mycobacterium smegmatis* (Gram-positive bacteria) after 24 h for bacteria and 48 h for yeasts. DMSO was used as negative control and nystatin, norfloxacin and vancomycin were used as positive controls for yeasts, Gram-negative and Gram-positive bacteria, respectively.

The results obtained (Table 2 and Figure 10) show that, in comparison with free tazobactam, both [Ag(I)-Tazo] and [Ag(I)-Tazo_2_] are more active against Gram-negative (*E. coli* and *P. aeruginosa*) and Gram-positive (*S. aureus MRSA*, *S. aureus*, *E. faecalis*, *M. smegmatis*) bacteria, with [Ag(I)-Tazo] being also more active against the *C. albicans* and *S. cerevisiae* yeasts, benefiting a possible synergistic effect between Ag(I) and tazobactam components (Alexander 2009; Lu et al., 2014).

**TABLE 2:** Determination of the minimum inhibitory concentration values (MIC, µg/mL) of the synthesized compounds, and the starting materials for *Candida albicans* and *Saccharomyces cerevisiae* (yeasts), *Escherichia coli* and *Pseudomonas aeruginosa* (Gram-negative bacteria) and *Staphylococcus aureus*, *Enterococcus faecalis* and *Mycobacterium smegmatis* (Gram-positive bacteria) after 24 h for bacteria and 48 h for yeasts.

Microorganisms		Ag_2_O	Tazobactam	[Ag(I)-Tazo]	[Ag(I)-Tazo_2_]	Positive control	Negative control
Yeasts	*C. albicans*	6.88	62.50	31.25	62.50	3.91 (Nys)	125.00
	*S. cerevisiae*	6.88	>62.50	31.25	>62.50	3.91 (Nys)	62.50
Gram-negative bacteria	*E. coli*	1.72	31.25	7.81	11.72	<0.49 (Nor)	62.50
	*P. aeruginosa*	1.66	15.63	0.98	0.98	<0.49 (Nor)	62.50
Gram-positive bacteria	*S. aureus MRSA*	6.88	62.50	7.81	31.25	0.98 (Van)	125.00
	*S. aureus*	13.75	31.25	15.63	15.63	3.91 (Van)	125.00
	*E. faecalis*	5.16	>125.00	15.63	31.25	<0.49 (Van)	125.00
	*M. smegmatis*	11.78	46.88	15.63	19.53	<0.49 (Van)	125.00

Legend: Positive controls: Nys—nystatin; Nor—norfloxacin; Van—vancomycin. Negative control: DMSO.

Note: The antimicrobial effect activity of tazobactam is limited by the effect of DMSO, for *S. cerevisiae* and *E. faecalis*.

Among the two Ag(I)-tazobactam frameworks, [Ag(I)-Tazo] is generally more active, with MIC values lower than [Ag(I)-Tazo_2_], except for *P. aeruginosa* and *S. aureus*, for which that both new compounds present the same MIC value.

From the list of microorganisms tested in this study, the yeasts were the least sensitive to tazobactam and its frameworks, with [Ag(I)-Tazo] being slightly more active.

Regarding the MIC values for the tested bacteria, generally, tazobactam and its silver derivatives are more efficient against Gram-negative bacteria. In fact, *P. aeruginosa* shown to be the most sensitive to tazobactam, [Ag(I)-Tazo] and [Ag(I)-Tazo_2_]. As previously reported, *P. aeruginosa* isolates were considered highly susceptible to the ceftolozane/tazobactam β-lactam–β-lactamase inhibitor combination, displaying MIC values lower than 4 μg/ml (Karlowsky et al., 2021). Both novel Ag(I) compounds unveiled herein also display very low MIC value (0.98 μg/ml) against this bacterium, showing a promising activity. The MIC values obtained for the new complexes are lower than not also tazobactam, but also lower that silver oxide itself.

Interesting results were obtained from the assay with two different strains of *S. aureus* (MRSA CIP 106760 and ATCC 25923). Both [Ag(I)-Tazo] and [Ag(I)-Tazo_2_] display higher activity than tazobactam against both strains, with an important note for the fact that [Ag(I)-Tazo] is even more active against the methicillin-resistant *S. aureus* (MRSA) strain than the *S. aureus* (ATCC).

It is worth noting that bacterial infections caused by both *P. aeruginosa* and *S. aureus* are commonly treated using β-lactams (Foster 2017; Gherardi et al., 2019). Meanwhile, the emergence of resistant strains to the usual therapy motivated the search for new ways to deal with these very important infections.

The obtained results highlight the importance of the synergy between tazobactam and silver metal centers that possess a well-recognized antimicrobial efficiency (Alexander 2009; Lu et al., 2014). Moreover, these striking, and very promising findings provide a sustainable and fast strategy to reuse the available antibiotics and obtain new and powerful ACFs to battle bacteria like *P. aeruginosa* and *S. aureus*, which are responsible for several nosocomial infections and highly capable to develop resistance mechanisms to the existing antibiotics (Foster 2017; Pang et al., 2019).

## Materials and Methods

### Reagents

The following reagents and solvents were purchased from commercial sources and used without further purification: tazobactam (C_10_H_12_N_4_O_5_S, Carbosynth Ltd.); silver(I) oxide (Ag_2_O, 99%, Alfa Aesar) and dimethyl sulfoxide (DMSO, 99%, Fluka).

### Synthesis

The new compounds [Ag_2_(µ_3_-Tazo)(µ_4_-Tazo)]_n_ (abbreviated as [Ag(I)-Tazo]) and {[Ag(µ-Tazo)(µ-TazoH)][Ag(µ-Tazo) (TazoH)]}_n_ (abbreviated as [Ag(I)-Tazo_2_]) were synthesized by reacting tazobactam (TazoH) and Ag_2_O in two different stoichiometries and using the liquid-assisted grinding (LAG) mechanochemical method in the presence of catalytic amounts of water for different periods of time (Table 3). The ball milling reactions were carried out in a Retsch MM400 ball mill at a frequency of 20 Hz, in 15 ml stainless steel snap closure jars containing two 7 mm stainless steel balls (approximately 2.7340 g). Both compounds could also be obtained by manual grinding and suspension methods, however more reaction time is needed as indicated in Table 3. The resulting powder was then dissolved in water, and colorless single crystals of [Ag(I)-Tazo] were grown by slow evaporation of the solvent after 3 weeks at room temperature. With respect to form [Ag(I)-Tazo_2_], the resulting powder was also dissolved in water and orange single crystals were grown by slow evaporation of solvent after 3 weeks at room temperature.

**TABLE 3:** Experimental conditions for the synthesis of compounds [Ag(I)-Tazo] and [Ag(I)-Tazo_2_].

Compound	Method	Tazobactam	Ag_2_O	Tazobactam:Ag ratio	Time	Solvent (H_2_O)	η (μL/mg)
[Ag(I)-Tazo]	Ball milling	144.7 mg	55.8 mg	1:1	5 min	100	0.499
		0.4806 mmol	0.2403 mmol				
	Manual grinding	72.3 mg	27.8 mg		15 min	100	1.00
		0.2403 mmol	0.1201 mmol				
	Suspension	72.2 mg	28.0 mg		48 h	2000	20.0
		0.2403 mmol	0.1201 mmol				
[Ag(I)-Tazo_2_]	Ball milling	167.7 mg	32.4 mg	2:1	5 min	100	0.500
		0.5583 mmol	0.1396 mmol				
	Manual grinding	83.9 mg	16.3 mg		10 min	100	1.00
		0.2792 mmol	0.0698 mmol				
	Suspension	83.8 mg	16.2 mg		48 h	2000	20.0
		0.2792 mmol	0.0698 mmol				

### Powder X-Ray Diffraction

Powder X-ray Diffraction data were collected in a D8 Advance Bruker AXS θ-2θ diffractometer (Bruker, Karlsruhe, Germany), equipped with a LYNXEYE-XE detector, copper radiation source (Cu Kα, λ = 1.5406 Å), operated at 40 kV and 30 mA, with the following data collection parameters: 3–60° 2θ range, step size of 0.02° and 1.5 s per step. The diffractograms were used to analyze the purity of compounds [Ag(I)-Tazo] and [Ag(I)-Tazo_2_] by comparing the experimental PXRD data with the patterns simulated from SCXRD data, using MERCURY 2021.2.0 (Macrae et al., 2008).

### Single Crystal X-Ray Diffraction

Crystals suitable for single X-ray diffraction studies were mounted on a loop with Fomblin^©^ protective oil. Data for [Ag(I)-Tazo] and [Ag(I)-Tazo_2_] was collected on a Bruker D8Quest diffractometer, with graphite-monochromated radiation (Mo Kα, λ = 0.71073 Å) at 293 K. X-ray generator was operated at 50 kV and 30 mA and APEX3 (Bruker 2016) program monitored data collection. Data were corrected for Lorentzian polarization and absorption effects using SAINT (Bruker 2014) and SADABS (Bruker 2014) programs. SHELXT 2014/4 (Sheldrick 2015b) was used for structure solution and SHELXL 2014/7 (Sheldrick 2015a) was used for full matrix least-squares refinement on *F* ^2^. These two programs are included in the WINGX-Version 2014.1 (Farrugia 1999; Farrugia 2012) program package. A full-matrix least-squares refinement was used for the non-hydrogen atoms with anisotropic thermal parameters. The H_CH_ were inserted in idealized positions and allowed to refine in the parent carbon atom. The hydrogen atoms of the COOH groups of [Ag(I)-Tazo_2_] were located from the electron density and inserted in idealized positions in the respective parent atoms and allowed to refine in the parent oxygen atom. MERCURY 2021.2.0 (Macrae et al., 2008) was used for packing diagrams and for polyhedral representation. PLATON (Spek and Anthony, 2018) was used for the determination of hydrogen bond interactions. Table 4 summarizes the data collection and refinement details for [Ag(I)-Tazo] and [Ag(I)-Tazo_2_]. Crystallographic data of compounds [Ag(I)-Tazo] and [Ag(I)-Tazo_2_] were deposited at the Cambridge Crystallographic Data Centre (Groom et al., 2016) (CCDC 2121840-2121841).

**TABLE 4:** Crystallographic data for compounds [Ag(I)-Tazo] and [Ag(I)-Tazo_2_].

	[Ag(I)-Tazo]	[Ag(I)-Tazo_2_]
Chemical formula	C_20_H_22_Ag_2_N_8_O_10_S_2_	C_20_H_23_AgN_8_O_10_S_2_
Formula weight	808.00	707.45
Crystal form, colour	Needle, colourless	Plate, colourless
Crystal size (mm)	0.20 × 0.04 × 0.04	0.16 × 0.10 × 0.05
Crystal system	Monoclinic	Monoclinic
Space group	*P*2_1_	*P*2_1_
*a* (Å)	12.4747 (13)	10.404 (3)
*b* (Å)	6.4407 (7)	25.480 (8)
*c* (Å)	16.9538 (16)	10.538 (3)
*β* (°)	110.961 (5)	109.793 (9)
*V* (Å^3^)	1272.0 (2)	2,628.5 (13)
Z	2	4
*d* (mg.cm^−3^)	2.126	1.788
*µ* (mm^−1^)	1.778	0.997
*θ* range (°)	3.266–26.493	2.080–28.833
Reflections collected/unique	10,530/4,978	24,760/12,766
R_int_	0.0487	0.0912
GoF	1.028	1.004
Final R indices^a^ ^,^ ^b^ [*I* > 2*σ*(*I*)]	*R*_*1*_ = 0.0380, w*R*_*2*_ = 0.0828	*R*_*1*_ = 0.0663, w*R*_*2*_ = 0.0873

a *R*_1_ = *Σ*||*F*_o_|—|*F*_c_||/Σ|*F*_o_|.

b *wR*_2_ = {Σ[*w* (*F*_o_^2^—*F*_c_^2^)^2^]/Σ[*w* (*F*_o_^2^)^2^]}^1/2^.

### Topological Analysis

Topological analysis of the obtained silver(I) coordination frameworks (Blatov 2006; Blatov et al., 2014) was performed by using a concept of underlying (simplified) net (O’Keeffe and Yaghi 2012; Li et al., 2014). Such networks were generated by reducing the ligands to the respective centroids while preserving their connectivity with silver(I) centers. Weak intermolecular interactions, such as hydrogen bonds, and argentophilic contacts, were not considered.

### Hot-Stage Microscopy

Hot-stage microscopy experiments were carried out using a Linkam TP94 device connected to a Linkam LTS350 platinum plate, using a 10°C/min heating rate. Images were collected, via the imaging software Cell D (Olympus United Kingdom Ltd., Hertfordshire, United Kingdom), with an Olympus SZX10 stereomicroscope. The observations were conducted under Fomblin^©^ oil with crystals that were previously indexed and whose cell parameters are similar to the previously determined crystal structures.

### Attenuated Total Reflection-Fourier Transform Infrared Spectroscopy

Fourier Transform Infrared Spectroscopy (FTIR) measurements were recorded on a Thermo Nicolet 6,700 spectrometer (Waltham, MA, United States) with attenuated total reflectance (ATR) mode by averaging 32 scans at a maximum resolution of 4 cm^−1^, registering the spectra at a wavelength interval of 4,000–650 cm^−1^.

### Elemental Analysis

Data were performed in a Fisons CHNS/O analyzer Carlo Erba Instruments EA-1108 equipment at the Instituto Superior Técnico Analyses Laboratory. Anal. Calculated for [Ag(I)-Tazo]: C, 29.5%; H, 2.72%; N, 13.76%; S, 7.87%. Found: C, 29.73%; H, 2.35%; N, 13.69%; S, 8.08%. Anal. Calculated for [Ag(I)-Tazo]_2_: C, 34%; H, 3.14%; N, 15.86%; S, 9.08%. Found: C, 34.39%; H, 2.98%; N, 15.82%; S, 9.16%.

### Differential Scanning Calorimetry and Thermogravimetry Analysis

Combined measurements were carried out on a SETARAM TG-DTA 92 thermobalance under a nitrogen flow with a heating rate of 10°C/min. The sample masses were in the range from 5 to 10 mg.

### Antimicrobial Activity Assays

The synthesized compounds and respective starting materials were tested against yeasts (*Candida albicans* ATCC 10231 and *Saccharomyces cerevisiae* ATCC 2601), Gram-negative (*Escherichia coli* ATCC 25922 and *Pseudomonas aeruginosa* ATCC 27853) and Gram-positive bacteria (*Staphylococcus aureus* (MRSA CIP 106760 and ATCC 25923), *Enterococcus faecalis* ATCC 29212 and *Mycobacterium smegmatis* ATCC 607) for the determination of their minimum inhibitory concentration (MIC) values. These values were determined by the microdilution method (Clinical and Laboratory Standards Institute 2018; Alves et al., 2020). Briefly, 100 μL of Mueller-Hinton (for bacteria) or Sabouraud Dextrose (for yeasts) liquid culture medium were added to all the 96-wells of the microtiter plates. Then, 100 μL of the testing compounds at a concentration of 1 mg/mL in DMSO were added to the first well. Serial dilutions of (1:2) were performed and 10 μL of bacterial inoculum was added to each well. The microtiter plates were incubated at 37°C for 24 and 48 h for bacteria and yeasts, respectively, and their growth was assessed through the analysis of the optical density of cultures at 620 nm using a Thermo Scientific Multiskan FC (Loughborough, United Kingdom) microplate reader.

## Conclusions

Two new Ag(I)-tazobactam coordination compounds were prepared by LAG, which revealed to be the most advantageous synthetic technique regarding time, yield and purity. The structural elucidation of the new compounds revealed a 3D metal-organic framework in [Ag(I)-Tazo] and an intricate combination of 2D+1D networks in [Ag(I)-Tazo_2_]. The obtained products are stable on shelf storage and up to 155°C, fundamental requisites for prospective pharmaceutical applications.

Despite the antimicrobial resistance to β-lactams, this class of antibiotics remains an important tool to treat infectious diseases. Importantly, the results disclosed herein show that both [Ag(I)-Tazo] and [Ag(I)-Tazo_2_] have greater antimicrobial activity than tazobactam alone, making it clear that if co-administered tazobactam were to be replaced by either MOF, the antimicrobial outcomes should be better. Nevertheless, this theory needs to be confirmed by *in vivo* and *in vitro* tests.

The coordination to the metal in both new compounds is established by the binding sites of tazobactam that are known to interact with enzymes (β-lactamases) in the human body and that may affect the interaction of tazobactam within the MOFs, leading to a different activity. Also, the potential release of labile silver cations certainly leads to synergistic effects promoting higher antimicrobial efficiency.

Furthermore, these results highlight the use of ACFs as a sustainable and valid alternative to circumvent the emergence of resistance mechanisms to the available antibiotics. As [Ag(I)-Tazo] and [Ag(I)-Tazo_2_] represent the first structurally characterized coordination compounds of tazobactam, this study also unveils new facets of tazobactam chemistry and will stimulate its application as a multifunctional linker for designing ACFs and related materials with higher antimicrobial efficiency.

## Data Availability

The datasets presented in this study can be found in online repositories. The names of the repository/repositories and accession number(s) can be found below: https://www.ccdc.cam.ac.uk/ CCDC 2121840-2121841.
