# Supplementary material for: Silver(I)-Tazobactam Frameworks with Improved Antimicrobial Activity
Source: Front Chem. 2022 Jan 25;9:815827. doi: 10.3389/fchem.2021.815827 (PMC8822216; doi:10.3389/fchem.2021.815827)
Supplement: Supplementary file 1 [file DataSheet1.docx]

Supplementary Material

Silver(I)-tazobactam frameworks with improved antimicrobial activity

Daniela R. Ferreira^1,2^, Paula C. Alves^1,2^, Alexander M. Kirillov^1^, Patrícia Rijo^3,4^, Vânia André^1,2^*

^1^Centro de Química Estrutural, Instituto Superior Técnico, Universidade de Lisboa, Lisboa, Portugal; ^2^Associação do Instituto Superior Técnico para a Investigação e Desenvolvimento (IST-ID), Lisboa, Portugal; ^3^Universidade Lusófona’s Research Center for Biosciences and Health Technologies (CBIOS), Lisboa, Portugal;^4^Research Institute for Medicines (iMed. ULisboa), Faculty of Pharmacy, Universidade de Lisboa, Lisboa, Portugal

*** Correspondence:** Vânia André, [vaniandre@tecnico.ulisboa.pt](mailto:vaniandre@tecnico.ulisboa.pt)

**1. STRUCTURAL ANALYSIS**


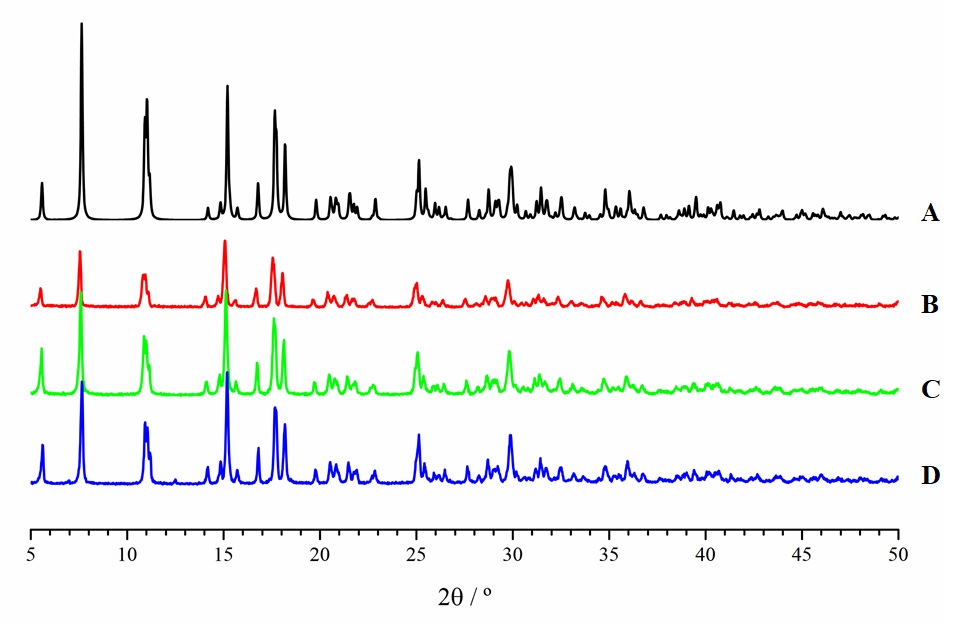


Supplementary Figure S1. PXRD diffractograms of compound [Ag(I)-Tazo]: simulated (A) and experimental obtained by the suspension method (B), experimental obtained by the manual grinding method (C), and experimental obtained by the ball milling method (D).


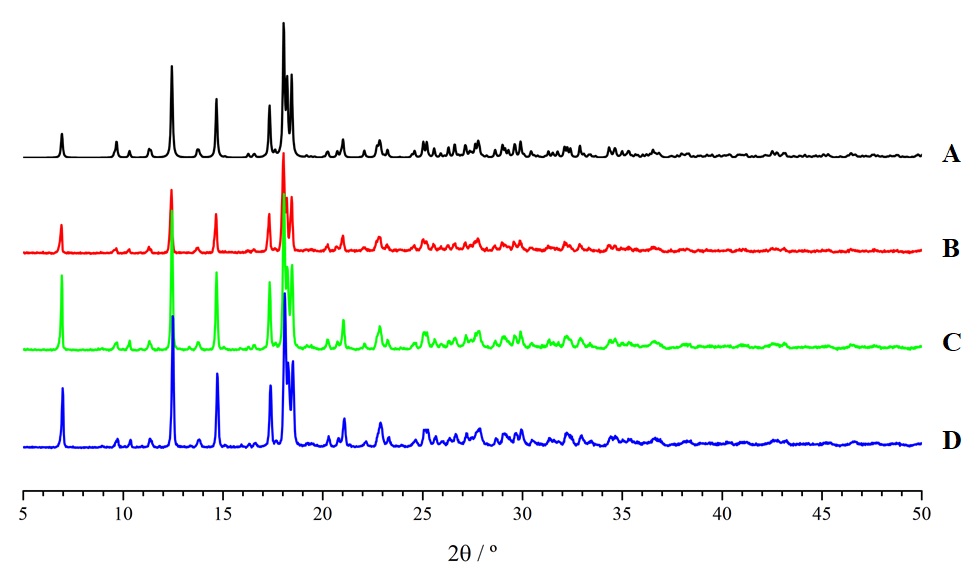


**Supplementary Figure S2.** PXRD diffractograms of compound [Ag(I)-Tazo_2_]: simulated **(A)**, experimental obtained by the suspension method **(B),** experimental obtained by the manual grinding method **(C)**, and experimental obtained by the ball milling method **(D)**.


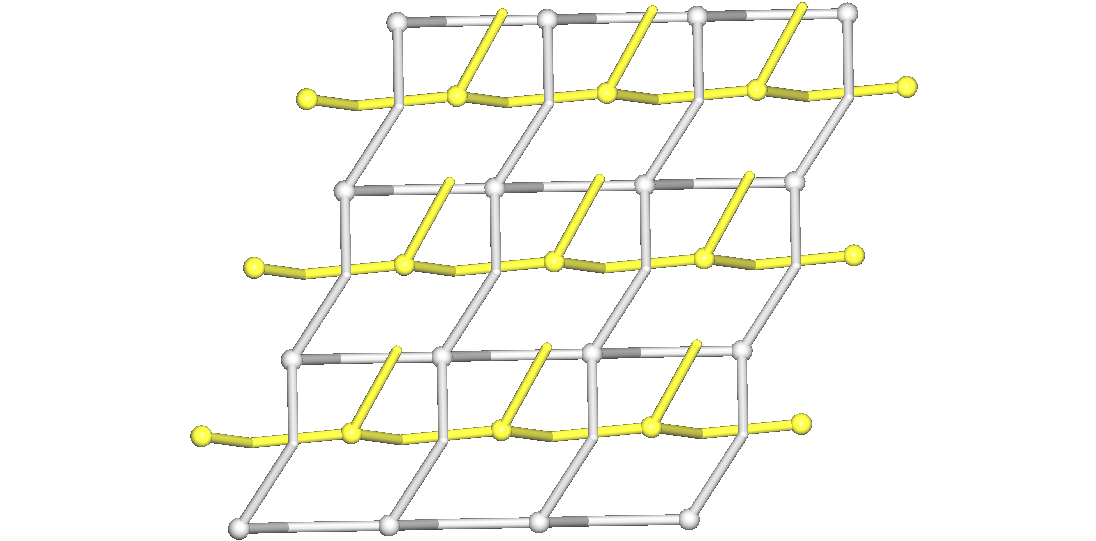


**Supplementary Figure S3.** Topological representation of the 2D+1D frameworks in [Ag(I)-Tazo_2_] wherein a 2D layer (gray) is decorated by 1D chains (yellow); rotated view along the *b* axis; Ag1 (gray) and Ag2 (yellow) centers are shown as balls.

**Supplementary Table S1.** Selected hydrogen bonds (Å) for [Ag(I)-Tazo] and [Ag(I)-Tazo_2_].

| **[Ag(I)-Tazo]** | | | |  | **[Ag(I)-Tazo_2_]** | | | |
| --- | --- | --- | --- | --- | --- | --- | --- | --- |
| Ag(1)-O(6) | 2.229(5) | N(5)-C(15) | 1.451(9) |  | Ag(1)-N(1) | 2.208(9) | O(7)-C(17) | 1.257(11) |
| Ag(1)-O(1) | 2.263(5) | N(5)-C(12) | 1.469(9) |  | Ag(1)-N(5) | 2.209(8) | O(15)-C(28) | 1.186(11) |
| Ag(1)-N(8)#1 | 2.281(5) | O(7)-C(11) | 1.248(10) |  | Ag(1)-O(6)#1 | 2.435(7) | O(1)-C(7) | 1.277(11) |
| Ag(1)-Ag(2)#2 | 3.0317(10) | O(7)-Ag(2)#2 | 2.297(5) |  | Ag(1)-O(2)#2 | 2.441(7) | N(12)-C(28) | 1.381(12) |
| Ag(2)-N(4) | 2.276(5) | N(1)-C(3) | 1.383(9) |  | Ag(2)-N(13) | 2.168(9) | N(12)-C(26) | 1.463(11) |
| Ag(2)-O(7)#3 | 2.297(5) | N(1)-C(2) | 1.459(8) |  | Ag(2)-N(9) | 2.176(9) | N(12)-C(30) | 1.464(12) |
| Ag(2)-Ag(1)#3 | 3.0317(10) | N(1)-C(5) | 1.476(9) |  | Ag(2)-O(12)#2 | 2.504(8) | N(4)-C(8) | 1.383(12) |
| S(2)-O(9) | 1.431(6) | N(7)-N(8) | 1.296(8) |  | S(2)-O(9) | 1.432(7) | N(4)-C(6) | 1.476(10) |
| S(2)-O(10) | 1.441(5) | N(3)-N(4) | 1.301(8) |  | S(2)-O(8) | 1.448(6) | N(4)-C(10) | 1.478(11) |
| S(2)-C(15) | 1.803(7) | O(2)-C(1) | 1.222(9) |  | S(2)-C(18) | 1.804(9) | O(10)-C(20) | 1.202(10) |
| S(2)-C(16) | 1.823(7) | N(8)-C(20) | 1.339(9) |  | S(2)-C(14) | 1.840(9) | N(7)-C(12) | 1.333(12) |
| S(1)-O(5) | 1.431(6) | N(8)-Ag(1)#4 | 2.281(5) |  | S(1)-O(4) | 1.433(7) | N(7)-N(6) | 1.335(11) |
| S(1)-O(4) | 1.436(6) | N(4)-C(10) | 1.340(8) |  | S(1)-O(3) | 1.440(6) | N(7)-C(13) | 1.475(10) |
| S(1)-C(5) | 1.792(6) | O(1)-C(1) | 1.269(9) |  | S(1)-C(10) | 1.806(10) | N(8)-C(20) | 1.386(12) |
| S(1)-C(6) | 1.835(7) | O(6)-C(11) | 1.236(10) |  | S(1)-C(4) | 1.823(9) | N(8)-C(16) | 1.470(11) |
| N(2)-C(9) | 1.335(8) |  |  |  | S(3)-O(14) | 1.435(6) | N(8)-C(18) | 1.472(11) |
| N(2)-N(3) | 1.339(7) |  |  |  | S(3)-O(13) | 1.437(7) | O(17)-C(37) | 1.267(13) |
| N(2)-C(8) | 1.452(8) |  |  |  | S(3)-C(30) | 1.784(10) | N(10)-N(9) | 1.316(11) |
| N(6)-C(19) | 1.334(9) |  |  |  | S(3)-C(24) | 1.848(10) | N(10)-N(11) | 1.318(11) |
| N(6)-N(7) | 1.339(7) |  |  |  | S(4)-O(18) | 1.436(7) | N(16)-C(40) | 1.406(13) |
| N(6)-C(18) | 1.468(8) |  |  |  | S(4)-O(19) | 1.442(8) | N(16)-C(38) | 1.455(12) |
| O(8)-C(13) | 1.183(9) |  |  |  | S(4)-C(38) | 1.802(11) | N(16)-C(36) | 1.463(12) |
| N(5)-C(13) | 1.394(9) |  |  |  | S(4)-C(34) | 1.826(10) |  |  |

**Supplementary Table S2.** Main non-covalent interactions in [Ag(I)-Tazo] and [Ag(I)-Tazo_2_]

| **[Ag(I)-Tazo]** | | | |  | **[Ag(I)-Tazo_2_]** | | | |
| --- | --- | --- | --- | --- | --- | --- | --- | --- |
| **Analysis of Short Ring-Interactions** | | | |  | **Analysis of Short Ring-Interactions** | | | |
| **Cg⋅⋅⋅Cg** | **Symm. Op.** | **Distance (Å)** |  |  | **Cg⋅⋅⋅Cg** | **Symm. Op.** | **Distance (Å)** |  |
| Cg(1)⋅⋅⋅Cg(1) | 2-X,-1/2+Y,1-Z | 4.813(5) |  |  | Cg(1)⋅⋅⋅Cg(8) | 1-X,-1/2+Y,-Z | 5.441(7) |  |
| Cg(5)⋅⋅⋅Cg(6) | X,-1+Y,1+Z | 4.478(4) |  |  | Cg(1)⋅⋅⋅Cg(11) | 1+X,Y,-1+Z | 5.155(8) |  |
| Cg(5)⋅⋅⋅Cg(6) | X,Y,1+Z | 3.681(4) |  |  | Cg(2)⋅⋅⋅Cg(1) | 2-X,1/2+Y,-Z | 5.716(7) |  |
| Cg(6)⋅⋅⋅Cg(5) | X,Y,-1+Z | 3.681(4) |  |  | Cg(2)⋅⋅⋅Cg(12) | 1+X,Y,-1+Z | 4.949(8) |  |
| Cg(6)⋅⋅⋅Cg(5) | X,1+Y,-1+Z | 4.178(4) |  |  | Cg(5)⋅⋅⋅Cg(7) | X,Y,Z | 4.468(6) |  |
|  |  |  |  |  | Cg(5)⋅⋅⋅Cg(11) | X,Y,-1+Z | 5.240(7) |  |
|  |  |  |  |  | Cg(6)⋅⋅⋅Cg(8) | X,Y,Z | 4.672(7) |  |
|  |  |  |  |  | Cg(6)⋅⋅⋅Cg(12) | 1+X,Y,Z | 5.627(7) |  |
|  |  |  |  |  | Cg(7)⋅⋅⋅Cg(2) | 1-X,-1/2+Y,-Z | 5.459(6) |  |
|  |  |  |  |  | Cg(7)⋅⋅⋅Cg(5) | X,Y,Z | 4.468(6) |  |
|  |  |  |  |  | Cg(7)⋅⋅⋅Cg(11) | X,Y,Z | 5.989(8) |  |
|  |  |  |  |  | Cg(8)⋅⋅⋅Cg(1) | 1-X,1/2+Y,-Z | 5.442(7) |  |
|  |  |  |  |  | Cg(8)⋅⋅⋅Cg(6) | X,Y,Z | 4.673(7) |  |
|  |  |  |  |  | Cg(8)⋅⋅⋅Cg(7) | 1-X,1/2+Y,1-Z | 5.229(8) |  |
|  |  |  |  |  | Cg(11)⋅⋅⋅Cg(5) | X,Y,1+Z | 5.240(7) |  |
|  |  |  |  |  | Cg(11)⋅⋅⋅Cg(6) | X,Y,1+Z | 4.639(7) |  |
|  |  |  |  |  | Cg(11)⋅⋅⋅Cg(7) | X,Y,Z | 5.990(8) |  |
|  |  |  |  |  | Cg(11)⋅⋅⋅Cg(8) | -X,-1/2+Y,1-Z | 5.797(8) |  |
|  |  |  |  |  | Cg(12)⋅⋅⋅Cg(5) | -1+X,Y,Z | 4.649(8) |  |
|  |  |  |  |  | Cg(12)⋅⋅⋅Cg(6) | -1+X,Y,Z | 5.627(7) |  |
| **Analysis of Y-X...Cg(Pi-Ring) Interactions** | | | |  | **Analysis of Y-X...Cg(Pi-Ring) Interactions** | | | |
| **X-Y⋅⋅⋅Cg** | **Symm. Op.** | **X⋅⋅⋅Cg (Å)** | **Y-X..Cg (⁰)** |  | **X-Y⋅⋅⋅Cg** | **Symm. Op.** | **X⋅⋅⋅Cg (Å)** | **Y-X..Cg (⁰)** |
| S1-O4⋅⋅⋅Cg(1) | X,Y,Z | 2.780(6) | 60.2(2) |  | S1-O4⋅⋅⋅Cg(1) | X,Y,Z | 2.863(9) | 59.6(3) |
| S1-O4⋅⋅⋅Cg(1) | X,Y,Z | 3.545(6) | 8309(2) |  | S2-O8⋅⋅⋅Cg(6) | X,Y,Z | 3.467(8) | 91.8(3) |
|  |  |  |  |  | S2-O9⋅⋅⋅Cg(2) | X,Y,Z | 2.872(8) | 59.1(3) |
|  |  |  |  |  | S3-O13⋅⋅⋅Cg(7) | X,Y,Z | 2.835(9) | 59.5(3) |
|  |  |  |  |  | S3-O14⋅⋅⋅Cg(11) | X,Y,Z | 3.405(9) | 89.9(3) |
|  |  |  |  |  | C28-O15⋅⋅⋅Cg(8) | 1-X,-1/2+Y,1-Z | 3.570(9) | 126.3(6) |
|  |  |  |  |  | C37-O1⋅⋅⋅Cg(6) | X,Y,Z | 3.430(11) | 121.4(7) |
|  |  |  |  |  | S4-O18⋅⋅⋅Cg(12) | X,Y,Z | 3.472(10) | 91.9(4) |
|  |  |  |  |  | S4-O19⋅⋅⋅Cg(8) | X,Y,Z | 2.735(10) | 59.8(3) |

**Supplementary Figure S4.** Two-dimensional fingerprint plots for the interactions in [Ag(I)-Tazo].

**Supplementary Figure S5.** Two-dimensional fingerprint plots for the interactions in [Ag(I)-Tazo_2_].


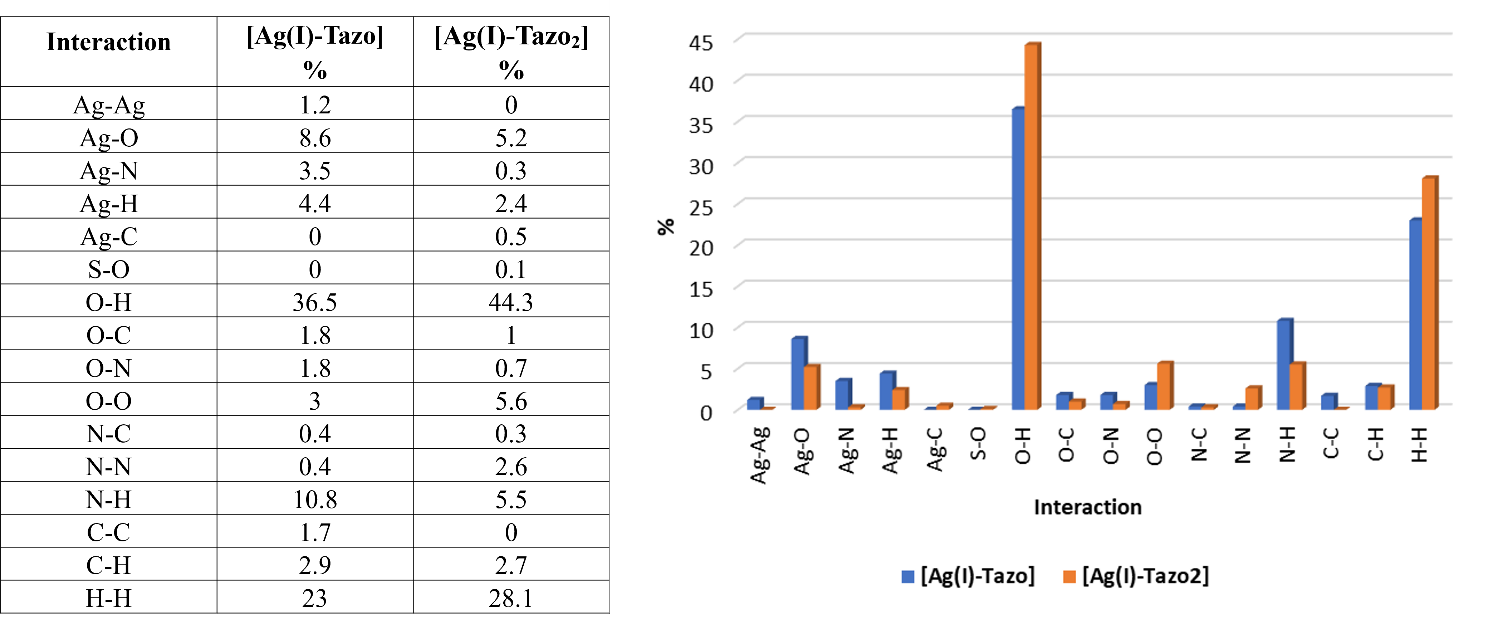


**Supplementary Figure S6.** Summary of the percentage (%) of the interactions taken from the 2D fingerprint plots for [Ag(I)-Tazo] and [Ag(I)-Tazo_2_].


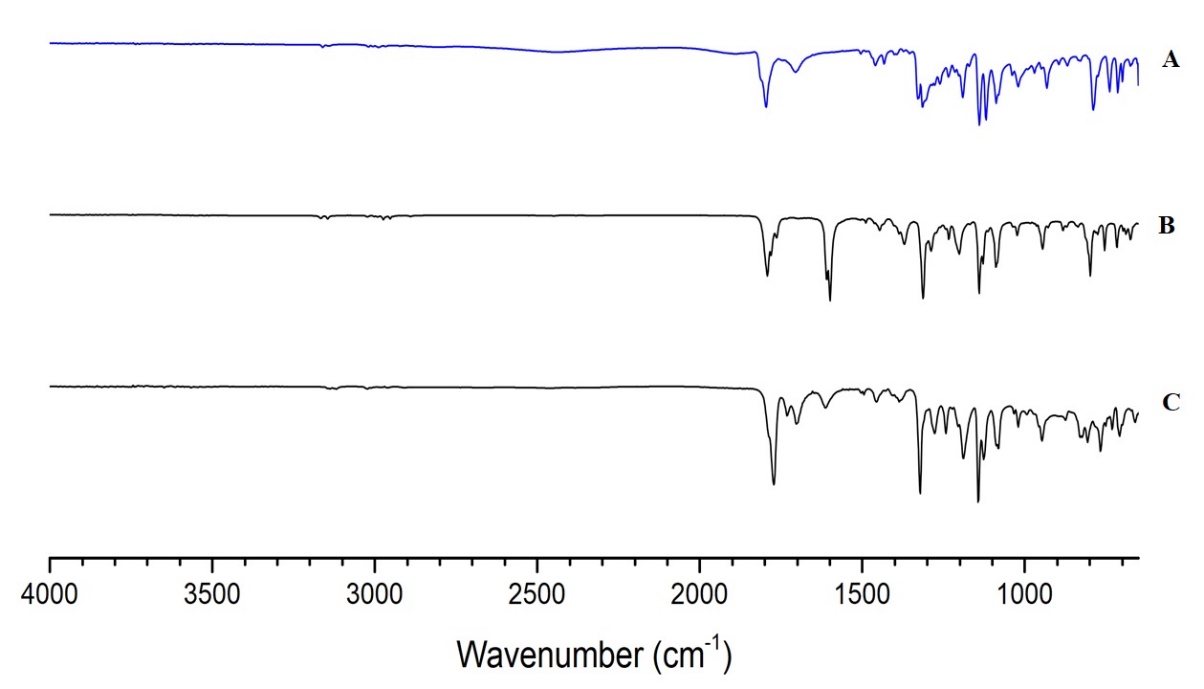


Supplementary Figure S7. Fourier Transform-Infrared/Attenuated Total Reflectance (FTIR-ATR) spectrum for pure tazobactam (A), [Ag(I)-Tazo] (B) and [Ag(I)-Tazo_2_] (C).

**Supplementary Table S3.** FTIR-ATR vibration modes of the interactions of some relevant peaks of tazobactam and [Ag(I)-Tazo] and [Ag(I)-Tazo_2_] compounds.

|  | **Wavenumber (cm^-1^)** | | |
| --- | --- | --- | --- |
| **Type of vibration** | **Tazobactam** | **[Ag(I)-Tazo]** | **[Ag(I)-Tazo_2_]** |
| C-SO_2_-C stretching | 1140, 1190, 1313 | 1139, 1206, 1311 | 1142, 1188, 1321 |
| N=N stretching | 1238, 1455 | 1233, 1446 | 1241, 1455 |
| Carbonyl group stretching from the amide | 1795 | 1791 | 1773 |
| Carbonyl group stretching from the carboxylic acid | 1702 | - | 1702 |
| Carboxylate group stretching | - | 1373, 1599 | 1384,1610 |

**2. STABILITY OF THE COMPOUNDS**

**
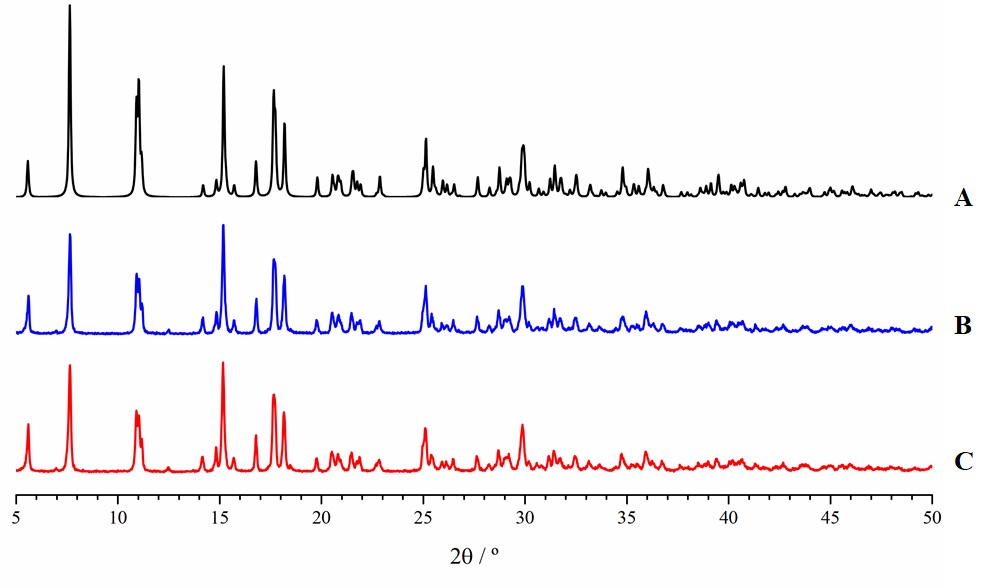
**

**Supplementary Figure S8.** PXRD diffractograms of compound [Ag(I)-Tazo]: simulated **(A)**, experimental obtained by the ball milling method **(B)**, and experimental obtained by the ball milling method after 5 months on shelf storage at room temperature **(C)**.


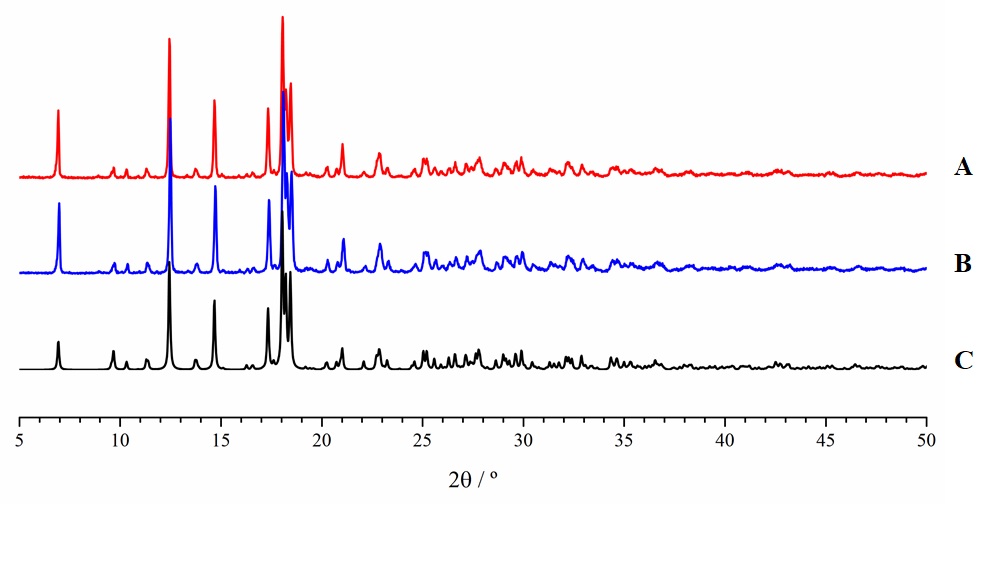


**Supplementary Figure S9.** PXRD diffractograms of compound [Ag(I)-Tazo_2_]: experimental obtained by the ball milling method after 5 months on shelf storage at room temperature **(A)**, experimental obtained by the ball milling method **(B)** and simulated **(C)**.


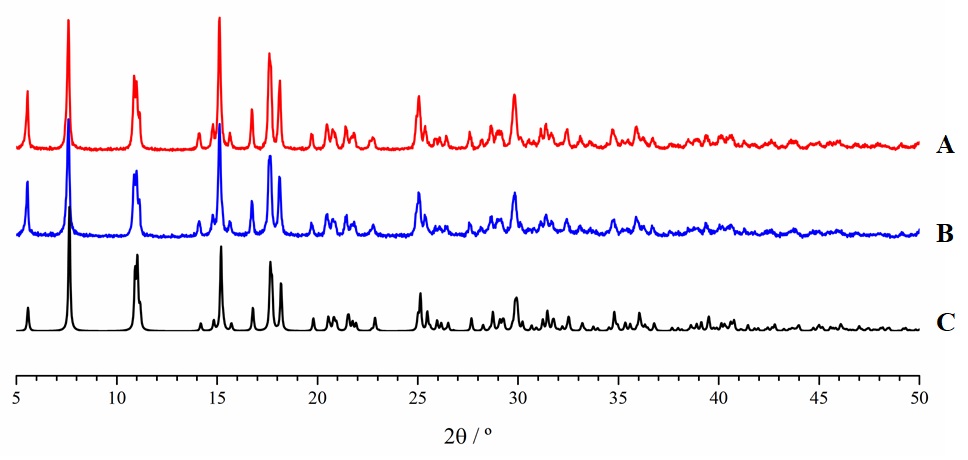


**Supplementary Figure S10.** PXRD diffractograms of compound [Ag(I)-Tazo]: experimental obtained by the manual grinding method after 5 months on shelf storage at room temperature **(A)**, experimental obtained by the manual grinding method **(B)** and simulated **(C)**.


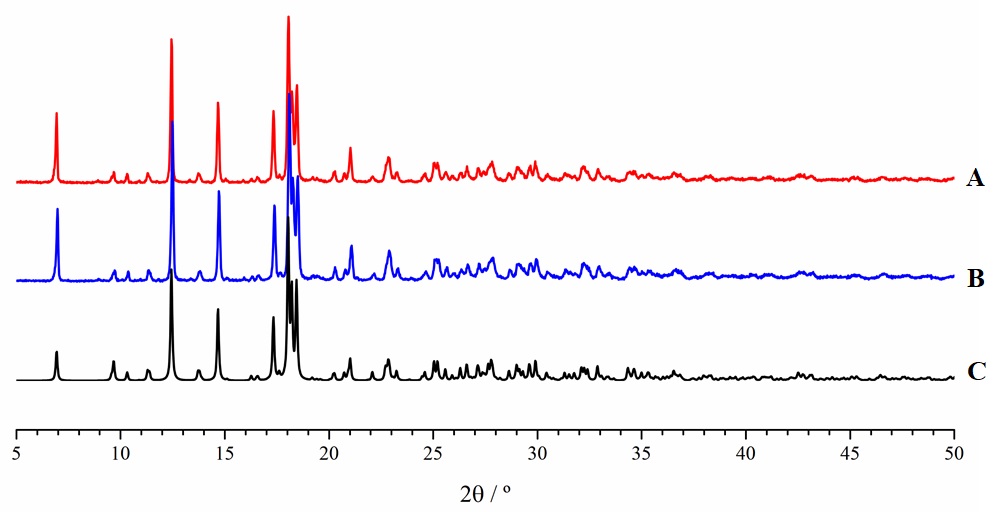


**Supplementary Figure S11.** PXRD diffractograms of compound [Ag(I)-Tazo_2_]: experimental obtained by the manual grinding method after 5 months on shelf storage at room temperature **(A)**, experimental obtained by the manual grinding method **(B)** and simulated **(C)**.


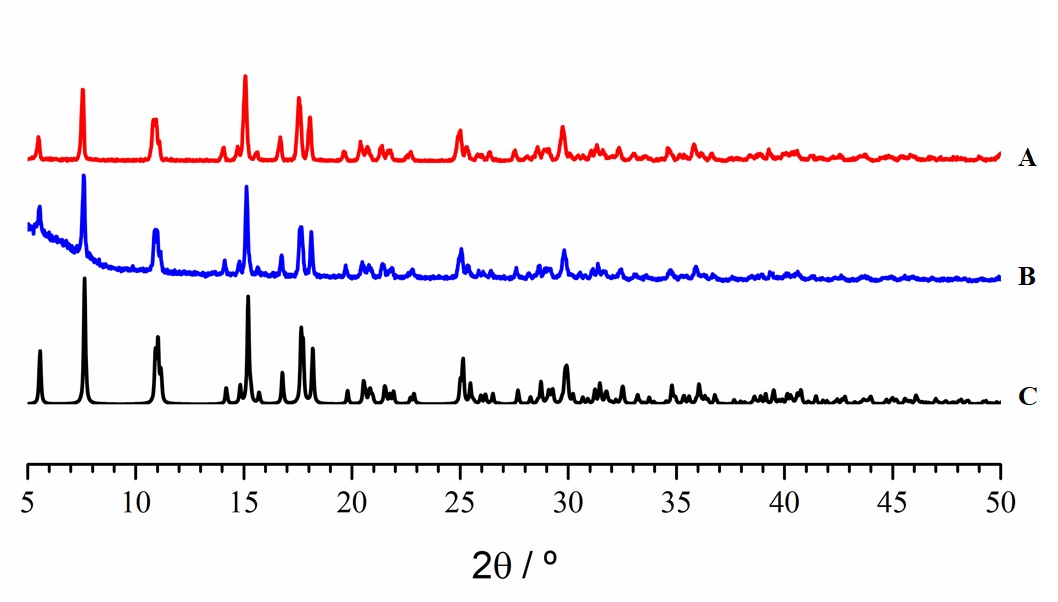


Supplementary Figure S12. PXRD diffractograms of compound [Ag(I)-Tazo]: experimental obtained by the suspension method after 5 months on shelf storage at room temperature (A), experimental obtained by the suspension method (B), and simulated (C).


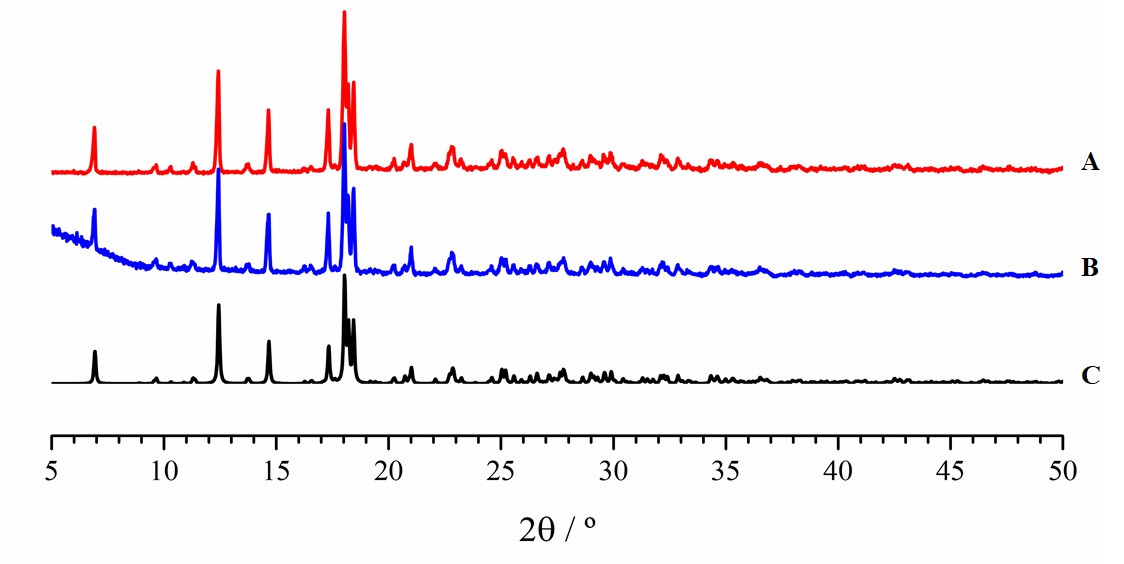


**Supplementary Figure S13.** PXRD diffractograms of compound [Ag(I)-Tazo_2_]: experimental obtained by the suspension method after 5 months on shelf storage at room temperature **(A)**, experimental obtained by the suspension method **(B)**, and simulated **(C)**.

**
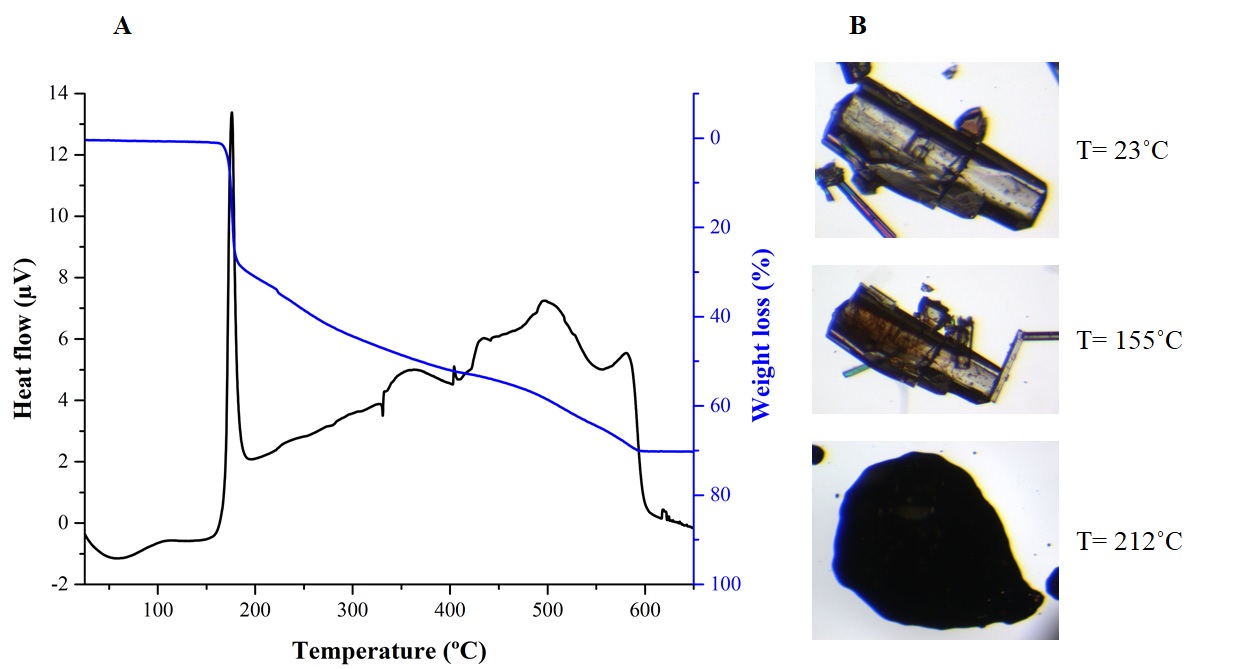
**

**Supplementary Figure S14. (A)** Differential scanning calorimetry (DSC) and thermogravimetry (TGA) and **(B)** hot-stage microscopy images with different temperatures for compound [Ag(I)-Tazo].
